# Intelligence

**Published:** 1829-01

**Authors:** 


### INTELLIGENCE.

### MONTHLY REPORT OF PREVALENT DISEASES.

The extraordinary mildness of the present season has given a character to the diseases which have prevailed. Dyspeptic complaints have been more than usually abundant, partly owing to the damp and close state of atmosphere, and partly, perhaps, to the circumstance, of those subject to indigestion having been less tempted to take the necessary proportion of air and exercise. Rheumatism in a chronic form has also been very common, and owing in a great measure to the same circumstances. Fever has likewise been met with, perhaps rather more frequently than usual; but the cases which have fallen under our own observation have not been of a severe character. On the other hand, the acute inflammations which usually present themselves about this period, have scarcely been seen, or, if so, only in individual instances.

*To the Editors of the Medical and Physical Journal.*

GENTLEMEN,—In the report of the trial of COOPER versus WAKLEY, there are some questions in the cross-examination of Mr. LAMBERT, which imply that I had delivered several demonstrations, and also a lecture, with the view of showing that Mr. B. Cooper's operation of lithotomy was unskilfully performed. As this imputation has been widely circulated through the medium of the press, I am desirous of refuting it in the most public manner, and I therefore trust you will give insertion to the following statement in your next number. In consequence of receiving many applications from gentlemen attending my lectures, who were subpœned on this trial, to describe to them the anatomy of the perinæum, I delivered a lecture, which was numerously attended, and among those present were several students who had been called on to give evidence, both by the plaintiff and defendant. In that lecture I pointed out the anatomical relations of the perinæum and neck of the bladder. I also gave several demonstrations in the dissecting room, in the usual routine, on the same parts. But in thus discharging what I considered the imperative duty of an anatomical lecturer, I studiously avoided all allusion to the operation performed by Mr. B. Cooper. On Saturday morning last I read the above report in the Morning Herald, and I felt anxious to have an opportunity of stating these facts in court. I therefore made an application through Sir A. Cooper to that effect, but I was informed by him that it was too late. Under these circumstances, I had no alternative but to publish the letter which appeared in the Morning Herald of Monday. In the evening of that day I received the following letter from Sir A. Cooper; and, by his kind permission, I insert a copy of it.

"*Conduit street, Dec.* 15, 1828.

"MY DEAR SIR,—You have done exactly as I wished, in publishing your letter. All the evidence on the part of the defendant had been examined, and I, who am ignorant of these matters, believed that it was impossible to retrograde. By sending your letter to the press you have completely exculpated yourself. Believe me, with real esteem,

Yours always, most truly,

ASTLEY COOPER."

In conclusion, I have only to add, that my colleague, Mr. PILCHER, has requested me to state, that, in the demonstrations which he has given, he has carefully abstained from all comment on the above operation.

I am, Sir, your obedient servant,

R. D. GRAINGER.

*Broad-street Buildings, Dec.* 16, 1828.

---

*Epidemic of Gibraltar.*—M. MOUREAU DE JONNES lately read, at the Royal Academy of Sciences in Paris, a paper on the yellow fever, which prevails at present at Gibraltar. According to the statements of this gentleman, the disease has been imported by a Swedish vessel, admitted after quarantine to discharge its cargo, although it had been refused admittance into the southern parts of Spain. This vessel arrived in August, but the fever was only communicated in September; and it was about the 6th of the month that the governor decided upon making known the infectious nature of the disease. From that period the usual senatory measures employed in such cases were taken throughout all Andalusia. This explanation of course assumes the contagious nature of the disease itself, which we should be inclined to doubt. We perceive, also, that a commission, consisting of three physicians, MM. Chervin, Trousseaux, and Louis, has been sent from Paris for the purpose of studying and reporting upon the nature of the disease.

We are happy to learn, that by the latest accounts, (dated Nov. 30,) a favorable change had occurred in the weather, and brought with it a mitigation of the epidemic.

No doubt is entertained of its being the same kind of epidemic as that which prevailed in 1803 and 1813-14. The symptoms are fever; severe pains across the loins; tenderness in the epigastrium; headache; great prostration of strength; suffusion of the eye; yellow tinge of the skin, and black vomit. The disease generally runs its course in from three to five days.

---

### SURGICAL PRACTICE AT GUY'S HOSPITAL.

*Cooper* v. *Wakley.*—This case, which was specially appointed for this morning, excited the most intense interest.

The defendant was charged with having published in the *Lancet*, a libel, imputing to the plaintiff, Mr. Bransby Cooper, the unskilful performance of an operation of lithotomy, which took place at Guy's Hospital in March last. The defendant pleaded several special pleas of justification, setting forth the matter charged as libellous, and averring that the whole of it was true.

The libel appeared in numbers 239 and 240 of the *Lancet*.

Sir James Scarlett, Mr. Pollock, Mr. Platt, Mr. Scarlett, were for plaintiff. The defendant pleaded his own cause, assisted by Mr. Brougham and Mr. Kelly. A long argument ensued between the defendant and the counsel for the plaintiff, as to who should first address the Court. The Lord Chief Justice consulted with his brother Judges in the Bail Court, and returned in about ten minutes, when he stated their opinions to be, that the task of beginning was upon the defendant.

Mr. Wakley then addressed the Jury. The report in the *Lancet* was not that of a supposed, but of a real operation; and was neither false, malicious, nor calumnious. Guy's Hospital was established not merely as a charity, but was also intended to be a great school for teaching surgery. It had been instituted by Mr. Guy, with an ample endowment, and 200,000*l.* had been subscribed to it. The funds belonging to the Hospital were immense, and it was of the utmost importance that it should be conducted on the best possible principles. Mr. Bransby Cooper had been elected Surgeon of that Hospital, and he was responsible to the public for the due execution of the duties of that important office, and had no right to complain, when his manner of performing his duties, as Surgeon of the Hospital, was freely canvassed before the public, provided it were done fairly and justly. The great injury was the injury done to the public, when incompetent men came forward and undertook these most serious and important duties; for how could they teach others to perform these operations skilfully, who were themselves unskilful? Mr. Wakley then called and examined his witnesses.

Mr. Holdiman Partridge, of Colchester—"I am a member of the College of Surgeons. I have been in practice more than fourteen years; have witnessed many operations in lithotomy, and have performed them myself sixteen or eighteen times. I witnessed the operation performed by Mr. Bransby Cooper at Guy's Hospital in March last. The report of that operation in the *Lancet* struck me at the time to be correct, and I have no reason to alter my opinion. The patient appeared a very healthy man. I think Mr. Cooper himself introduced the staff; but the second incision was made without the staff. After the first external incision, all instruments were withdrawn. The hands of the patient were tied to his feet, and his knees to his neck, as represented by the model now produced. The patient remained in that position nearly an hour. During that period a sound was repeatedly introduced. Several cuts were attempted to be made into the bladder with a knife. This instrument (a cutting gorget) was introduced into the wound.

A blunt gorget was also introduced, and the scoop and several pair of forceps. During the operation the patient called out several times to the operator to desist. The operator stated several times that he could not explain the difficulty. I cannot say that I think Mr. Cooper performed the operation in a scientific manner. I do not think that it was performed in such a manner as the public have a right to expect from a surgeon of Guy's Hospital. The average time for performing operations of this description is four or five minutes. The operation in question occupied, I think, nearly an hour."

In his cross-examination by Sir James Scarlett, the witness stated his opinion, that Mr. Callaway, who assisted in the operation, was a man of great skill. Where the stone lay above the pubis, witness had always successfully extracted it in the way ultimately adopted by Mr. Cooper. Witness was asked, whether he thought the words in the libel, "the knife was carried onwards *somewhere*," did not convey an idea that the knife did not go into the bladder? He thought it meant that it might or might not have entered. He would neither contradict or affirm that the forceps were a second time used with considerable force.

Mr. John Clapham witnessed the operation; and the report in the *Lancet* was correct, as far as he recollected.

Cross-examined.—He should think Mr. Callaway a competent judge of operations of this kind. Witness admitted that he had made a false representation of his age to the Company in obtaining his certificate. Lord Tenterden instantly stopped his evidence, and witness withdrew.

Mr. Joachim Gilbert, a member of the College of Surgeons, witnessed the proceedings of Mr. Cooper for about thirty-five minutes, and he could not endure to stay any longer. The operator used great and unnecessary violence. He passed the forceps four times following after he had failed to extract the stone, passed his finger into the wound, and then poked a pair of crooked forceps about in it. After passing them three times, he called for "Sir Astley's knife," and made a cut with it, and passed his finger into the wound; and in so doing used violence, twisting the finger about in the wound.

Cross-examined.—Witness is assistant to Mr. Phelps, who married the defendant's sister. The first incision made was rightly performed. Witness never performed the operation of lithotomy; but had witnessed at least fifteen operations of the kind at Guy's Hospital. The operation of lithotomy requires greater skill than tying the subclavian artery. A very ignorant surgeon might, by accident, tie the subclavian artery with success.

Mr. John Thomas saw part of the operation. His testimony respecting it was similar to that of the last witness.

Mr. Jeffry Pearl saw the operation performed. The report in the *Lancet* was correct, except that Mr. Cooper asked for "Sir Astley's knife," and not for "my uncle's knife," as stated in the report. There was no gush of urine as usual, but merely a trickling. [The witness was examined to various minute facts, deposed to by some of the preceding witnesses, and, in part, corroborated their testimony. He also spoke to the violence used as described by those witnesses, and stated, that three fingers at once were introduced.]

Cross-examined.—Witness never performed lithotomy himself. Mr. Lambert introduced him to the defendant. At that interview, the defendant asked witness whether the report was correct. The defendant and Mr. Lambert endeavoured to show that the forceps had passed between the bladder and the rectum.

Mr. James Lambert stated that he furnished the report to the defendant. The witness then described the circumstances attending the operation nearly in the words of the libel; after which he described the appearances of the body of the patient after death. I do not think Mr. Cooper a good operator, but I once saw him tie the subclavian artery in a very skilful manner. That is not a difficult operation to a man who has any nerve. I do not think Mr. Cooper's abilities are adequate to the office of surgeon to Guy's Hospital.

This witness underwent a long cross-examination. He was admitted a surgeon three years ago. Derived considerable emolument from contributing to the *Lancet*, but not more than from his own profession. Had once an angry altercation with Mr. Cooper. He had been refused admission to Guy's

Hospital and St. Thomas's on account of the report. Was expelled from Middlesex Hospital four years ago, because he then became connected with the *Lancet*.

Mr. Alexander Lee was the next witness. He considered the report correct, but written very unprofessionally. No surgeon of experience would venture to give an opinion without speaking to the operator. Next to the operator the person most competent to give an opinion is the assistant-surgeon. The mode of operating for the stone is not settled in any country, and any surgeon uses what instruments he pleases.

On his cross-examination, the witness said he was a clerk, and had been a potatoe merchant some years ago.

Mr. Bolton, a pupil, gave evidence similar to that of the preceding witnesses.

Mr. Harrison, treasurer to Guy's Hospital.—Mr. Cooper was elected assistant-surgeon, and Sir Astley Cooper consulting-surgeon, on the 14th May, 1825. Mr. Cooper was demonstrator under his uncle, and gave great satisfaction. He was recommended by all the surgeons in the Hospital. Sir A. Cooper did not know that the Hospital intended to elect his nephew, till I informed him of it. I knew that Mr. Cooper had served in Norwich Hospital, and also as army-surgeon in Spain, under the Duke of Wellington. He likewise served in the same capacity in Canada, at the close of the American war. He afterwards studied at Edinburgh for two years. He then came to Guy's Hospital. Mr. Cooper has always maintained the reputation which induced the Hospital to elect him.

Mr. Wakley then proposed to put in the preparations taken at the Hospital, as part of his case. The preparations were then brought into Court, and were examined by several medical gentlemen.

Mr. Holdiman Partridge, examined.—"I have examined these preparations. Whilst they are in the glass I cannot see the incisions. I see the opening in the bladder. I cannot give any reason why the operation should have lasted an hour, without having the preparations in my hand; and I would not like to give a decisive opinion on the subject without having examined them by myself."

Mr. Wakley said that his case was now closed.

Sir James Scarlett addressed the Jury for the plaintiff. The attack in the *Lancet*, he contended, was one of the most injurious that ever falsehood or malice invented. The plaintiff was, as it were, put upon his trial, and the gentlemen of the Jury could scarcely be aware what it was that the plaintiff had to complain of, unless, indeed, they might have read it in the Evening Papers of yesterday, in which it was given with a curious exactitude, which could only be accounted for by supposing that it was furnished either by the defendant or his attorney. Thus, according to the forms of law, the plaintiff, who is the party complaining, even upon the threshold of that Court, the sanctuary of justice, meets with the dagger of the assassin, and before he can utter a cry of complaint, the weapon is struck deeper and deeper into his side. The learned counsel then gave a sketch of the professional career of Mr. B. Cooper in Spain and Canada, and contended that his education and experience were such as to justify his being chosen as surgeon to Guy's Hospital. The learned counsel dwelt largely on the opportunities which the plaintiff had, under the auspices of Sir A. Cooper, of acquiring the most intimate knowledge of the most abstruse parts of his profession. Sir A. Cooper would never have had the plaintiff as his assistant had he not been convinced of his capability, for then he would have risked his own character in his private practice. From this it might fairly be presumed that no private motives of friendship could have led to the plaintiff's being thus engaged, and that it could be only his skill which led to such an employment. The libel intimated that Mr. Cooper was chosen surgeon of the Hospital because he was the nephew of Sir A. Cooper; but the defendant, by his witness, Mr. Harrison, had proved the very reverse. He should call the other surgeons of the Hospital, who, abandoning all spirit of rivalry, would come forward to save from the ruin which such a libel would heap upon him, a gentleman whose skill and experience were of the highest order. The learned counsel then characterised the editor of the *Lancet* to be, according to his own showing, a literary depredator, living upon five or six thousand a year, and riding in his carriage, by means of resources which were drawn from others. Mr. Cooper, who is now thirty-four years of age, had performed many operations, and it should be remembered that his successful ones had not been reported. There was a time when the operation of lithotomy was attended with the greatest danger, but the improved modes of operation had lessened the mortality from an average of one in four to one in seven and a half. Mr. Cooper had performed the operation upon a person eighty-seven years of age with complete success. Sir James then described the operation, and said that Lambert had himself made the opening between the bladder and the rectum, as he should prove by the evidence of those medical gentlemen who had seen the parts immediately prior to Lambert's exclaiming that there was a breach in that part, and at which time there was no opening whatever. The learned counsel then read the libel, and commented upon it as he went along; and then concluded a speech which occupied more than three hours. Before entering upon the evidence, Sir James said that Clapham, who had yesterday stated in his testimony that *he* had not sworn that he was twenty-one years of age when he obtained his certificate, had actually so sworn, as he could prove; and Lambert, who had sworn that he did not know that Clapham was applying for his certificate, had actually signed a certificate of moral character to enable him to obtain it.

The publication of the libels was then admitted, and they were read by the officer of the Court.

THOMAS CALLAWAY examined by Mr. Pollock.—" I am Assistant Surgeon to Guy's Hospital. I have been seventeen years in the profession, and have seen nearly all the operations which have taken place in that hospital. It is usual for one of the surgeons to assist, and on the occasion of Mr. Cooper's operation I assisted. The operation lasted about fifty minutes. I could not see the first incision from the position in which I stood, but I felt Mr. Cooper cut into the grove of the staff which I had in my hand, and have therefore no doubt but that the bladder was penetrated. I was present at the *post-mortem* examination, and then found no reason to doubt that the first incision penetrated the bladder. No one can so well tell the difficulties of an operation as the operator: the assistant would not possess better means of judging than a spectator. There was a difficulty in this case, owing to the situation of the stone. Do not know that the forceps reached the bladder; but no man would be justified in introducing them, unless he was convinced that he had reached the bladder with the first incision. I have no doubt but that the forceps entered the bladder after the first incision. The situation of the stone satisfactorily accounted for the forceps not finding it. Generally the stone is to be found in the inferior part of the bladder, in the hollow of the pelvis; but here it was in the anterior part of the bladder. If the opening had been already large enough, the cutting gorget could do no injury; but if the opening was not large enough, it would make it so. In the result Mr. Cooper extracted the stone; and I think he used proper means, considering its position. No great or unnecessary violence was used, nor unnecessary instruments. I think the operation was performed with as much care as the difficulties of the case could have received. The delay was occasioned entirely by the situation of the stone, and the difficulty of detecting it. I think Mr. B. Cooper a skilful surgeon. I attended the post-mortem examination. There is a cellular substance between the rectum and the bladder, and I saw nothing which could induce me to think that the forceps had penetrated that substance. If any such injury had been done, I should have discovered it by the extravasation of blood."

Some anatomical preparations were then brought into Court, and Mr. Wakley proceeded to cross-examine the witness upon one of them (the bladder) after it was removed from the glass for that purpose. The examination was entirely as to the anatomy of the parts, in order, if possible, to show that no difficulty ought to have existed in finding the stone.

The Witness proceeded.—" The enlargement of the opening might take ten seconds. The cutting gorget was used only once. Have threatened to expose the corrupt system of election at the Hospital. Never said that Mr. Cooper was fitter to spend a large fortune than to be surgeon of the Hospital. Might have said, like other disappointed candidates, that I ought to have been elected. (*A laugh.*) I have seen the appearance produced in the cellular membrane by the forceps passing between the rectum and the bladder; but, in the cases in which I have witnessed these appearances they were by no means similar to the present. In the former cases there was extravasated blood; in the latter there was none, though the cellular membrane was dark coloured."

Mr. C. A. Key deposed that he is the senior surgeon. Had seen several operations performed by Mr. B. Cooper; and that Mr. Cooper did not lose in these operations more than the average number of patients. From the description of the difficulty, as given by Mr. Calloway, witness thought he should himself have pursued the same course as that which had been adopted by Mr. Cooper. The length of time during which the operation lasted, was no criterion as to the skill of the operator. If any violence had been used, the cellular membrane would have been found lacerated, and in a state of slough, with extravasated blood. "According to my experience, it is only in a few cases that you can reach the bladder with the finger; but it is desirable to do so if possible."

Joseph Laundy deposed that he attended operations at Guy's Hospital for upwards of thirty years, and he held, on this occasion, the instruments for Mr. Cooper. Has witnessed operations in lithotomy that lasted as long. Those operations were performed by Mr. Travers, Mr. Green, and Mr. Cline, sen. One by Mr. Cline, sen. occupied one hour and forty minutes. Remembers Sir A. Cooper being once above an hour performing the operation.

Dr. Hodgkin, professor of morbid anatomy at the Hospital, deposed that he examined the parts after death, and there was no appearance of wound between the bladder and the rectum. The kidneys were mottled with a white substance, such as is frequently the case in similar diseases. When Lambert asked to see the parts, he was shown them in the presence of witness. Lambert pointed out the opening between the bladder and the rectum. Witness immediately charged him with having made the opening, which he denied; but witness thinks, if the opening had been there before, it could not have escaped him. The opening must have been made after death; for if before there must have been extravasated blood. He thought that Mr. Cooper was a fair surgeon and a very good anatomist.

Mr. Brodie deposed, that he has met Mr. B. Cooper in private practice, and has conversed with him upon surgical topics; and he should think, from what he has seen of him, that he is a very intelligent surgeon; has heard Mr. Callaway's description of the manner in which the operation was performed, and thinks it was a difficult operation, and performed with skill.

Cross-examined.—Remembers the lecturers' meeting in 1825, at the Freemason's Tavern, when the lectures were first published; it being deemed a common cause, there was a proposition to pay Mr. Abernethy's expenses if he would move for an injunction against the defendant. Witness paid part of Mr. Abernethy's expenses upon his second application.

Re-examined.—"It appeared to me to be a great grievance to have the lectures published, because they were published incorrectly, and brought great discredit on those whose lectures were published."

Mr. Travers.—"I have been in practice twenty years, and I have been in the profession since 1800; I have heard the evidence of Mr. Calloway as to the operation, and I have heard of no circumstance which could impeach the skill of the operator. I think the operator is the best judge as to the instrument which ought to be used. The length of time is no criterion of the skill of the operator. I am acquainted with Mr. Cooper, and think that he is an ingenious and intelligent surgeon, and fit for the situation he holds as surgeon of Guy's Hospital. There are often cases of lithotomy which for a long time baffle the skill of the best operator, and I conceive the case in question to be one of that description.

Mr. Green was next examined.—"I am the nephew of the late Mr. Cline, and have been for eight years surgeon to St. Thomas's Hospital. I have often performed the operation of lithotomy, and am reputed to be very successful. I witnessed one capital operation by Mr. Bransby Cooper, which he performed admirably well. The operation was that of tying the external iliac artery, which, for skill, is somewhat like putting a ligature on the subclavian artery. From Mr. Calloway's description of the operation, I think it was skilfully conducted, and I should conceive it to have been a case of great difficulty; the length of time is of itself no criterion of the skill of the operator, the most skilful surgeon might have occupied the same time, and yet the result have been equally fatal."

Cross-examined.—"It requires great anatomical knowledge to tie the iliac artery. I was at the meeting at the Freemason's Tavern. Some lectures of mine at that time had been published. I contributed to the expenses of the injunction."

Re-examined.—"The lectures were incorrectly published, only one of them was free from misrepresentation."

Dr. Babington and Dr. Roget testified to the skill of Mr. Cooper.

Sir A. Cooper.—"I was subpœnaed by the defendant, and I heard the account given by Mr. Harrison of the education of Mr. Bransby Cooper. That account was perfectly correct. Mr. Bransby Cooper, when he first went to the Hospital, was a good anatomist and a very good surgeon, but he wanted experience in hospital practice. Still a young man must not be crushed in the outset, or the country will be deprived of perhaps first-rate abilities."

Cross-examined by Mr. Wakley.—"Sir Astley, have you never said that a good surgeon ought to be like a good general, that he should wade up to the neck in blood? Have you not said so in your lectures to your pupils?"

Sir A. Cooper.—"I don't know; I may have said so; I am sometimes fond of using strong expressions. I like, in short, to be understood. (*Great laughter.*) I think it one of the greatest evils that a man should be attacked in early life; and that he should be crushed, because he happened to have one misfortune, however capable he might be to practise in his profession. It is a power which I think the public press ought not to have." (*Laughter.*)

Mr. Wakley.—"Sir Astley, do you not think that the interests of the public would be best consulted by having men of experience appointed as surgeons to the hospitals, and not to appoint them for the purpose of acquiring experience?"

Sir Astley.—"I think that every Hospital ought to have an assistant-surgeon, who would thereby be prepared for the situation which he would subsequently hold."

Mr. Wakley.—"Was Mr. Bransby Cooper an assistant before he became surgeon?" Sir Astley Cooper.—"No, he was not; but I think it would be a good regulation to adopt in all of them."

Mr. Wakley.—"What was the particular difficulty in this case?" Sir A. Cooper.—"There was so little water in the bladder, that this man must have made water immediately before the operation. If the bladder were full of water the stone would have been easily struck."

Mr. Wakley.—"What is the danger to be guarded against in the operation of lithotomy?"

Sir A. Cooper.—"The chief danger to be guarded against is violence."

Mr. Wakley.—"Supposing the stone to be felt, but enveloped in the folds of the bladder, would not the more prudent course be, after having vainly tried for ten minutes to extract it, to place the patient in his bed again?"

Sir A. Cooper.—"I should certainly think not, if the stone could be felt. In that case the surgeon should persevere until he got it away."

Mr. Wakley.—"Have you not read Celsus, Sir Astley?" (*Laughter.*)

Sir A. Cooper.—"I have dipped into Celsus, but I do not consider Celsus as a good surgical authority, although he is a classical one." (*Much laughter.*)

Mr. Wakley.—"Is it not the practice in Edinburgh and Paris to put the patient to bed in such cases as I have supposed?"

Sir Astley Cooper.—"I have studied in Edinburgh, and I have witnessed operations of lithotomy in Paris, but I never saw such a practice resorted to."

Mr. Wakley.—"How long may the contraction of the bladder continue?"

Sir Astley.—"It might last an hour."

Mr. Wakley.—"What evil could result by placing the patient in bed until the contraction was over?"

Sir Astley.—"Just this—What you would not like—You would have two operations instead of one to perform. *(Laughter.)* If the stone was felt, the surgeon ought to persevere."

Mr. Wakley.—"Why is it more difficult to introduce the finger after death than before?"

Sir Astley.—"If you had ever put your finger into the bladder of a living subject you would know the difference. By putting your finger into the bladder of the dead subject you would have known as much of the difficulty of this case as you do now of what is passing in the moon." *(Much laughter.)*

Reexamined by Sir J. Scarlett.—"My nephew was a surgeon in the army, and he was my demonstrator of anatomy."

Mr. Dalrymple, of Norwich, deposed to Mr. Cooper's general character as a surgeon.

Mr. Watson put in the affidavit of Clapham, one of the defendant's witnesses, by which it appeared that he had misrepresented his case.

Sir James Scarlett.—"That is my case."

Mr. Wakley proceeded to reply. After referring to the origin and progress of the *Lancet*, the defendant declared that the proceedings of that day were the most peculiar that ever took place. One would have supposed, from the parade with which this case was heralded, that the report in the *Lancet* was a fabrication—that no such operation was ever performed—and that it had no foundation from beginning to end, except in the malevolent mind of the defendant. One of the witnesses for the plaintiff admitted that there were 200 spectators; and yet, with all the influence of Mr. B. Cooper, he was only able to produce one single spectator to rebut the charge made against him. The plaintiff sought every where but in the proper place for witnesses; he had surgeons from St. Thomas's Hospital, he had Sir Astley Cooper from Conduit-street, and it was to be wondered that the Emperor of Morocco was not also thrust into the box. With reference to the influence which Sir Astley Cooper had in the appointment of surgeons to Guy's Hospital, he put it to the jury whether Sir Astley was entitled to recommend any man to the care of patients as he had done his own nephew in this instance. This hospital belonged not to the directors, but to the poor, for whose use public funds had been granted. Mr. Callaway was elected an assistant surgeon, and Sir Astley Cooper consulting surgeon, on the same day that Mr. Bransby Cooper was appointed to the situation of surgeon. It was a saying of John Hunter, that bad surgeons made work for good ones; and that good surgeons would starve but for bad ones. Perhaps it was in reference to the operations of Mr. B. Cooper that these two supernumeraries have been appointed. Could it be pretended that Mr. Cooper was a skilful surgeon when he was unable to explain the causes of the difficulty that presented itself? Could he (Mr. Cooper) be said to have had presence of mind when, whilst he was plunging his instruments into the unfortunate patient, he openly declared that he could not find the reason for not being able to extract the stone. The poor man repeatedly called on them and implored them to loose him—he knew the torture of his complaint, he felt the torture which he was then undergoing, still he cried to be let go.—"No," said Mr. B. Cooper, "my reputation is concerned —I must take out the stone, even though you should die." He did die. The defendant most solemnly protested to God that he had no ill will towards Mr. Cooper, he believed him to be a most amiable private character; but as a surgeon—as a surgeon of a public hospital—as a public functionary, he denounced him to be ignorant and incompetent. Let the jury weigh well the evidence, and if they felt that they, if afflicted in a similar way, would not suffer Mr. Bransby Cooper to operate on them, then they must find their verdict for the defendant.

His lordship then proceeded to sum up the evidence.

After an absence of two hours, the jury returned a verdict for the plaintiff, damages 100*l.*

### METEOROLOGICAL JOURNAL,

*From November 20th, to December 20th,* 1828.

By Messrs. HARRIS and Co. Mathematical Instrument Makers, 50, High Holborn.

| November | Rain gauge. | Moon. | Thermom. | | | Barometer. | | De Luc's Hygrom. | | Winds. | | Atmospheric Variations. | | |
|---|---|---|---|---|---|---|---|---|---|---|---|---|---|---|
| | | | 9 A.M. | MAX. | MIN. | 9 A.M. | 10 P.M. | 9 A.M. | 10 P.M. | 9 A.M. | 10 P.M. | 9 *a.m.* | 2 *p.m.* | 10 *p.m.* |
| 20 | | | 44 | 54 | 44 | 30.01 | 29.94 | 65 | 62 | WNW | W | Fine | Fine | Cloudy |
| 21 | | ○ | 48 | 57 | 50 | 29.84 | .80 | 63 | 65 | W | W | Fine | —— | Fine |
| 22 | | | 52 | 56 | 41 | .72 | .81 | 66 | 69 | WSW | WNW | Fine | Cloudy | Show'ry |
| 23 | .07 | | 45 | 56 | 43 | .90 | .87 | 66 | 67 | SW | SW | —— | —— | Fine |
| 24 | | | 46 | 52 | 45 | .61 | .66 | 67 | 70 | SW | SW | —— | Fine | Fine |
| 25 | | | 50 | 55 | 49 | .80 | .83 | 69 | 70 | SW | SW | Foggy | Fine | Fine |
| 26 | | | 54 | 59 | 48 | .76 | .73 | 71 | 70 | WSW | W | Fine | Cloudy | Show'ry |
| 27 | .12 | | 50 | 58 | 46 | .83 | 30.00 | 68 | 67 | WNW | WNW | Fine | Fine | Show'ry |
| 28 | | | 50 | 59 | 45 | .95 | .. | 68 | 67 | W | WNW | Cloudy | Fine | Cloudy |
| 29 | | ☾ | 49 | 59 | 50 | 30.00 | .. | 66 | 66 | W | WNW | Fine | Fine | Fine |
| 30 | | | 54 | 56 | 49 | .. | 29.90 | 65 | 63 | WNW | NW | Cloudy | —— | Cloudy |
| Dec. | | | | | | | | | | | | | | |
| 1 | | | 51 | 51 | 35 | 29.87 | 30.05 | 62 | 56 | NNW | ENE | Fine | Fine | Fine |
| 2 | | | 36 | 50 | 36 | 30.31 | .22 | 54 | 57 | SE | S | —— | —— | —— |
| 3 | | | 40 | 51 | 41 | .05 | .02 | 56 | 63 | W | W | —— | —— | Cloudy |
| 4 | | | 50 | 53 | 43 | .10 | .11 | 67 | 67 | WNW | WNW | —— | —— | Cloudy |
| 5 | | | 52 | 53 | 42 | .06 | 29.94 | 67 | 64 | W | SSW | Foggy | Cloudy | Cloudy |
| 6 | | | 45 | 52 | 45 | 29.80 | .65 | 95 | 67 | SSW | SW | Fine | Fine | Rain |
| 7 | | ● | 50 | 53 | 44 | .45 | .40 | 66 | 65 | W | SW | —— | —— | —— |
| 8 | | | 45 | 48 | 39 | .38 | .14 | 66 | 66 | W | WNW | Foggy | Rain | —— |
| 9 | .71 | | 40 | 48 | 38 | .45 | .80 | 65 | 64 | W | WNW | Foggy | Fine | Fine |
| 10 | | | 39 | 54 | 44 | 30.02 | 30.00 | 64 | 66 | W | W | Sl. Fog | Fine | Cloudy |
| 11 | | | 54 | 56 | 43 | 29.79 | .17 | 67 | 64 | W | WNW | Rain | Fine | Fine |
| 12 | .10 | | 45 | 53 | 42 | 30.24 | .24 | 65 | 70 | W | W | Fine | Fine | Cloudy |
| 13 | | ☽ | 48 | 54 | 48 | .21 | .26 | 68 | 66 | W | W | Cloudy | Cloudy | —— |
| 14 | | | 49 | 50 | 40 | .30 | .11 | 67 | 65 | W | SW | Fine | Fine | —— |
| 15 | | | 41 | 50 | 40 | .15 | .08 | 64 | 66 | S | S | Foggy | Fine | Fine |
| 16 | | | 48 | 51 | 46 | .00 | 29.87 | 68 | 67 | WSW | WSW | Fine | —— | —— |
| 17 | | | 49 | 58 | 55 | 29.71 | .73 | 71 | 74 | W | W | Rain | Fine | Rain |
| 18 | | | 56 | 56 | 43 | .83 | .40 | 72 | 78 | W | WNW | Show'ry | Show'ry | Rain |
| 19 | .55 | | 46 | 52 | 45 | .61 | .83 | 65 | 64 | WNW | NW | Fine | Fine | Fine |

The quantity of Rain fallen in the month of November, was 1 inch and 2.100ths.

